# Neurogenic tachykinin mechanisms in experimental nephritis of rats

**DOI:** 10.1007/s00424-020-02469-z

**Published:** 2020-10-17

**Authors:** Kristina Rodionova, Karl F. Hilgers, Eva-Maria Paulus, Gisa Tiegs, Christian Ott, Roland Schmieder, Mario Schiffer, Kerstin Amann, Roland Veelken, Tilmann Ditting

**Affiliations:** 1 Department of Internal Medicine 4 (Nephrology und Hypertension), Friedrich-Alexander University Erlangen, Loschgestraße 8, 91054 Erlangen, Germany; 2 Center of Internal Medicine, University Medical Center Hamburg-Eppendorf, Hamburg, Germany; 3 Department of Internal Medicine 4 (Nephrology und Hypertension), Paracelsus Private Medical School, Klinikum Nuremberg, Nuremberg, Germany; 4 Department of Nephropathology, University of Erlangen, Erlangen, Germany

**Keywords:** Renal innervation, Nephritis, Tachykinins, Dendritic cells, Sympathetic nerve activity

## Abstract

We demonstrated earlier that renal afferent pathways combine very likely “classical” neural signal transduction to the central nervous system and a substance P (SP)–dependent mechanism to control sympathetic activity. SP content of afferent sensory neurons is known to mediate neurogenic inflammation upon release. We tested the hypothesis that alterations in SP-dependent mechanisms of renal innervation contribute to experimental nephritis. Nephritis was induced by OX-7 antibodies in rats, 6 days later instrumented for recording of blood pressure (BP), heart rate (HR), drug administration, and intrarenal administration (IRA) of the TRPV1 agonist capsaicin to stimulate afferent renal nerve pathways containing SP and electrodes for renal sympathetic nerve activity (RSNA). The presence of the SP receptor NK-1 on renal immune cells was assessed by FACS. IRA capsaicin decreased RSNA from 62.4 ± 5.1 to 21.6 ± 1.5 mV s (*p < 0.05) in controls, a response impaired in nephritis. Suppressed RSNA transiently but completely recovered after systemic administration of a neurokinin 1 (NK1-R) blocker. NK-1 receptors occurred mainly on CD11^+^ dendritic cells (DCs). An enhanced frequency of CD11c^+^NK1R^+^ cell, NK-1 receptor^+^ macrophages, and DCs was assessed in nephritis. Administration of the NK-1R antagonist aprepitant during nephritis reduced CD11c^+^NK1R^+^ cells, macrophage infiltration, renal expression of chemokines, and markers of sclerosis. Hence, SP promoted renal inflammation by weakening sympathoinhibitory mechanisms, while at the same time, substance SP released intrarenally from afferent nerve fibers aggravated immunological processes i.e. by the recruitment of DCs.

**Electronic supplementary material:**

The online version of this article (10.1007/s00424-020-02469-z) contains supplementary material, which is available to authorized users.

## Introduction

Effects of renal sympathetic nerve fibers on renal excretory function are very well documented [4, 11], but the role of sympathetic efferent as well as primary afferent sensory neurons in the inflammation and immune responses in the kidney is far less understood [58]. A role of renal nerve ablation in patients with renal disease and hypertension is so far not worked out [57]. Of note, in spite of putatively solid experimental evidence, it was far from straightforward to prove the clinical benefit of renal nerve ablation for the treatment of hypertension over the last decade [5, 25, 56, 57]. Hence, the physiology of renal autonomous innervation might be still far more complex than so far envisioned.

Renal innervation comprises not only efferent sympathetic but also afferent peptidergic nerves [11]. The situation is further complicated by the dual role of afferent renal innervation: on the one hand, afferent renal nerve fibers are said to control efferent sympathetic nerve activity [11]. On the other hand, afferent nerve fibers are known for a long time to release neuropeptides like CGRP and SP influencing local circulation and interfering with immune processes [8, 12].

The influence of autonomous innervation on acute and chronic inflammation in the respiratory and gastrointestinal tract including liver, the skin, the joints, and even the kidney is well documented [1, 51, 55, 58]. All components of the immune system and of visceral organs are innervated by the autonomous nervous system [24, 39]. In the kidney, the vessels, the glomeruli, the tubules, juxtaglomerular cells, and the renal pelvis are innervated by efferent sympathetic and afferent peptidergic nerves [32, 48, 58].

Immune cells like macrophages and dendritic cells (DCs) are abundant in healthy kidneys and renal inflammation. In all forms of glomerulonephritis, a role of macrophages in initiation of renal disease is well established [22, 53]. Additionally, over the last decades, robust evidence was found that renal DCs as specific antigen presenting cells play a pivotal role in the pathogenesis of inflammatory processes in renal disease [9, 14, 50].

Immune cells like T cells, macrophages, and DCs express receptors for neurotransmitter and neuropeptides like SP [20, 34, 54]. SP-positive nerve fibers were detected in the vicinity to afferent and efferent arterioles and glomeruli, and macrophages and DCs were detected in close vicinity to primary sensory neurons in the kidney, where they would offer an excellent environment for neuroimmunomodulation in health and disease [47, 58].

SP belongs to the mammalian tachykinin family [19, 36] and is one of the most important neuropeptides during neurogenic inflammation [16] involved in allergic asthma, chronic bronchitis, intestinal inflammation, pancreatitis, immune-mediated hepatitis, arthritis, cutaneous inflammation, and resistance to infection under experimental conditions [3, 10, 26, 29].

Since afferent renal innervation subserves a sympathodepressory reno-renal reflex [30], it seemed possible that neuronally secreted SP could also modulate this effect during stimulation of renal afferent nerves. We provided evidence that SP from afferent renal nerves is involved in a sympathoinhibitory system via a neurokinin 1-receptors (NK1-R)–dependent mechanism since NK1-R are the natural binding site of SP [13]. Interaction between efferent sympathetic and afferent renal nerves via this SP-dependent mechanism is presumably not restricted to the kidney: Neuropeptides like SP might be secreted along the whole surface of the axon and not only at the nerve endings [58] so that release of SP from afferent axons along their putative pathway through sympathetic ganglia could modulate renal sympathetic nerve activity (RSNA) [13].

Hence, we wanted to test the hypothesis that alterations in SP dependent mechanisms of renal innervation are involved in experimental mesangioproliferative glomerulonephritis in a complex manner. We assumed that the sympathoinhibition via a NK1-R-dependent mechanism would be impaired, whereas neurogenic release SP from afferent renal nerves intrarenally should have proinflammatory consequences. Eventually, renal innervation would aggravate experimental nephritis due to altered neural SP activity.

## Materials and methods

### General procedures

#### Animals

Procedures were performed in male Sprague-Dawley rats (Charles River, Sulzfeld, Germany) weighing 250–300 g in accordance with the National Institutes of Health (NIH) Guide for the Care and Use of Laboratory Animals and approved by the local government agency (Regierung von Mittelfranken, Ansbach, Germany).

#### Induction of anti-Thy1.1 nephritis

Anti-Thy-1.1 nephritis was induced by injection of 1.75 mg/kg OX-7 monoclonal antibody (mAb) into the tail vein [58].

#### Assessment of urinary protein excretion

Rats were kept in metabolic cages 24 h, 48 h, and 6 days after antibody administration for determination of urinary albumin and urinary protein excretion during 24 h. The rat albumin ELISA Quantitation Kit (Bethyl Laboratories, Montgomery, USA) was set up using Nunc-Immuno 96-well flat-bottom high-binding Maxisorb-polystyrene microtiter plates (Nunc, Roskilde, Denmark) to quantitatively measure levels of urinary albumin [58].

#### Administration of inhibitors

The chronic effect of the neuropeptide SP was analyzed by pretreatment of rats with 5 mg/kg of the neurokinin receptor 1 (NK1-R) antagonist aprepitant (a generous gift from Merck & Co) i.p. 12 h and 1 h before OX-7 injection and every 24 h after antibody administration. NK1-R is the physiological binding site of SP. Control animals were administered with vehicle or aprepitant administration without concomitant OX-7 injection.

#### Anesthesia

For all procedures, animals were initially anesthetized with a mixture of O_2_, 50% N_2_O, and ~ 1.5% Isofluran® and subsequently methohexital (800 μg/kg/min, IV) [13].

### In vivo experiments

#### Arterial and venous lines

Arterial femoral catheters were connected to a transducer (Stratham P23Db) to record arterial blood pressure (MAP) and heart rate (HR). Femoral venous lines were used for administration of substances [13].

#### Renal artery catheter [13]

The tip of a PE-10 catheter (OD 0.61 mm, ID 0.28 mm) was stretched by heat to an OD of 120 μm over 10 mm length and heat coiled around a glass rod with a diameter of 1.2 mm. The coil was cut so that a 90° curve with a radius of 0.6 mm was left. It was inserted into a vascular introducer sheath made from an intravascular infusion catheter (22G, Insyte-W, Becton Dickinson, Germany). The system was inserted into the left femoral artery and advanced up the aorta to position to the ostium of the left renal artery. The left kidney was exposed via a left lateral incision and carefully retracted ventrally, while the rat was lying on its right side. The curved micro-tip catheter was manipulated to advance the tip 1–1.5 mm into the left renal artery to then be used for the injection of substances.

#### Recording of RSNA

Recordings of right-sided RSNA were performed as previously described [13, 31]. A renal nerve bundle was dissected free from connective tissue and placed on a bipolar electrode (0.2-mm stainless steel wire, Science Products, Frankfurt, Germany). RSNA was recorded from a proximal right-sided renal nerve branch, and RSNA signals were amplified 50,000 times and filtered (1 kHz low pass; 100 Hz high pass) using a band pass amplifier (CyberAmp 320 with an AI402x50 Ultra Low Noise Differential amplifier; Axon Instruments, Foster City, CA, USA).

The signal was channeled to an A/D oscilloscope (HM 305–3; Hameg, Frankfurt, Germany) and an audio amplifier (AM8 audio monitor; Grass-Telefactor, West Warwick, RI, USA) for visual and auditory evaluation. RSNA signal quality was assessed by its pulse synchronous rhythmicity and by the decrease due to ganglionic blockade by trimetaphan camsylate (10 mg/kg IV; Arfonad®; Roche, Basel, Switzerland). Eventually, the nerve bundle was fixed to the electrode using silicone adhesive (Bisico S4i; Bielefelder Dentalsilicone, Bielefeld, Germany).

The nerve signals were full-wave rectified and integrated over 1-s intervals using a commercially available data acquisition and analysis software (SciWorks 7.2, DataWave Technologies, Loveland, CO, USA).

#### RSNA responses to intrarenal TRPV1 stimulation with capsaicin in anti-Thy1.1 nephritis rats and controls

Groups of rats either suffering from nephritis or controls were instrumented as follows: arterial and venous catheters for recording of blood pressure and for fluid and drug administration; left-sided electrode for RSNA recording; renal artery catheter for putative intrarenal chemical stimulation of SP-dependent sympathoinhibitory mechanisms via afferent peptidergic nerve fibers with the TRPV1 receptor agonist capsaicin as previously reported [13]. In short, sympathetic nerve activity was decreased by administration of 4 bolus injections of the TRPV1 agonist capsaicin every 15 min into the kidney via the renal artery. As previously described, after the intrarenal administration of capsaicin, there was always only a brief increase in afferent renal nerve activity, while sympathetic activity continued to decline [13] (see also supplemental material: section B). Therefore, this afferent sympathoinhibitory mechanism from the kidney has been termed “neuroparacrine.” Although the sympathoinhibitory activity does not finally recover to its initial levels until after several hours, sympathoinhibition could nevertheless be broken at any time briefly and completely, if a substance P antagonist was administered intravenously. This suggest that the stimulation of TRPV1 receptors by capsaicin is followed by a considerable release of SP from afferent renal nerve fibers inside and also outside the kidneys.

RSNA recording experiments commenced with baroreceptor loading and unloading, by IV bolus administration of the α_1_-agonist methoxamine (10 μg) and the vasodilator Na^+^-nitroprusside (NIP 1 μg), respectively, in randomized order with a recovery period of 15 min. The NK_1_-receptor antagonist RP67580 (10^−2^ M; 10 μl) [49] was given IV before and after administration of increasing doses of capsaicin (3.3 × 10^−7^ M, 6.6 × 10^−7^ M, 1.0 × 10^−6^M, 3.3 × 10^−6^ M, 10 μl each, 1 dose/15 min) into the renal artery as mentioned above. These doses are known to be not effective intravenously [13].

#### Inhibition of SP effects by NK1-receptor blockade

In acute experiments, NK1-R blockade (NK1 receptors are the physiological binding site of SP) was achieved with the potent and highly selective NK1-R antagonist RP67580 as mentioned above [49]. For ease of use, we administered for chronic NK1-R blockade 5 mg/kg of the NK1-R antagonist aprepitant (a generous gift from Merck & Co) i.p. 12 h and 1 h before OX-7 injection and every 24 h after antibody administration [23]. Control animals were administered with vehicle or aprepitant administration without concomitant OX-7 injection.

### Ex vivo procedures

#### Sampling of material

In anesthetized rats, kidneys were removed after perfusion with saline. Portions of renal cortex were snap-frozen in nitrogen for mRNA analysis, fixed in methyl Carnoy’s solution for histology or embedded in tissue-embedding medium. The second kidney was processed for flow-cytometric analysis.

#### Immunohistochemistry

After overnight fixation in methyl Carnoy’s solution, tissues were dehydrated by bathing in increasing concentrations of methanol, followed by 100% isopropanol. Embedded in paraffin, 2 μm sections were cut with a Leitz SM 2000 R microtome (Leica Instruments, Nussloch, Germany). After deparaffinization, endogenous peroxidase activity was blocked with 3% H_2_O_2_. A mouse monoclonal antibody detecting proliferating cells (PCNA) (Dakocytomation; Glostrup, Denmark) was used as well a mouse monoclonal anti-ED1 antibody (Serotec, Düsseldorf, Germany) for detection of macrophage. Staining reactions were carried out with a 0.1% diaminobenzidine tetrahydrochloride/0.02% H_2_O_2_ detection system (Vector Laboratories, Burlingham, California, USA).

#### Isolation of leukocytes from kidneys as previously described [2]

Briefly, kidneys were passed through 100 μm nylon mesh with RPMI 1640. The cells were hydrated with medium supplemented with 5% newborn calf serum. The cell suspension was centrifuged, the pellets resuspended with 37% Percoll solution (Amersham-Biosciences, Freiburg, Germany), and centrifuged again. Leukocytes were stained using a standard protocol including pre-blocking of Fc receptors.

#### Flow-cytometric analysis

For detection of DCs, a FITC-labeled anti-rat CD11c antibody (Serotec, Düsseldorf, Germany), and for detection of macrophages, an anti-rat ED1 antibody (Serotec, Düsseldorf, Germany) and a FITC-labeled sheep-anti-mouse-IgG (1:100; Dianova, Hamburg, Germany) were used. To investigate the distribution of NK-1R on immune cells, a rabbit-anti-human-NK-1R antibody (1:2.000; Novus Biologicals, Littleton, CO, USA) and a PE-labeled donkey-anti-rabbit-IgG was used (Jackson, Newmarket, Suffolk, UK). Data were analyzed using a FACScan Flow Cytometer (BD Biosciences) and Cellquest Software.

#### Immunocytochemistry of NK1-R

For NK1-R immunostaining, renal cryostat sections (10 μm thick) on glass slides were fixed in acetone/methanol (1:1) and blocked with PBS containing 3% BSA and 10% rabbit normal serum. Subsequently, slides were incubated with a primary antibody raised against the amino terminus region of human NK-1R (Santa Cruz Biotechnologies, Heidelberg, Germany) and specific markers for macrophages (mouse anti-rat ED1; Serotec, Düsseldorf, Germany) as well as DCs (mouse-anti-rat CD11c antibody; Serotec, Düsseldorf, Germany). Eventually, binding sites of NK-1R were detected using Cy3-conjugated rabbit-anti-goat antibody (1:500; Jackson ImmunoResearch/Dianova). Detection of ED1 and CD11c was performed using an Alexa488-conjugated donkey-anti-mouse antibody (Molecular Probes, Invitrogen, Karlsruhe, Germany).

#### Immunocytochemistry for SP

For detection of SP-positive sensory nerve fibers, immunocytochemistry for SP (rabbit-anti-SP, Peninsula, 1:1000) was utilized. Briefly, 15 μm cryostat sections from formaldehyde perfusion fixed rat kidneys were incubated with the primary antibody dissolved in TBS containing 1% BSA and 0.5% Triton X100 followed by incubation with donkey-anti-rabbit Alexa 555 (Molecular Probes, 1:1000) dissolved in TBS for 1 h at room temperature. Sections were investigated in a Biorad MRC 1000 confocal system attached to a Nikon Diaphot 300 inverted microscope. The yellow (568 nm) and blue (488 nm) lines of a krypton-argon laser were used for excitation of Alexa 555 and green autofluorescence of kidney tissue, respectively. Merged two channel confocal images were adjusted for contrast and brightness using Adobe Photoshop.

#### Western blot analysis

Renal cortex was homogenized in lysis puffer containing 0.5% (v/v) NP40 (Nonidet P40), 137 mM NaCl, 2 mM EDTA, 50 mM Tris/HCl, pH 7, and 10% (v/v) glycerol. Following centrifugation, supernatants were stored at − 80 °C. For western blot analysis, 30 μg protein was fractionated by SDS/10% PAGE and blotted on to a nitrocellulose membrane. For detection of TNFα, the antibody was purchased from Genzyme (IP-400). A HRP-conjugated goat-anti-rabbit antibody and a POD-conjugated rabbit-anti-goat antibody (Dianova, Hamburg, Germany) were used as secondary antibodies. Western blot was developed using an ECL® system (Amersham Bioscience, Freiburg, Germany). Semiquantitative evaluation was performed using the Gel Doc 2000 System (Bio-Rad Laboratories, München, Germany).

#### Real-time RT-PCR detection of mRNA

Total RNA was isolated from renal cortical tissue using the NucleoSpin RNA ΙΙ Isolation Kit (Macherey-Nagel, Düren, Germany). Total RNA was transcribed using SuperScript ΙΙ RNase H^−^ reverse transcriptase, oligonucleotides, and oligo (dT) primers from Invitrogen (Karlsruhe, Germany). Real-time RT-PCR was performed using a LightCycler system and LightCycler-FastStart DNA Master SYBR-Green Ι Mix (Roche Diagnostics). Primer sequences were 5′ rat β-actin GCC TTC CTT CCT GGG TAT G, 3′ rat β-actin TCA GGA GGA GCA ATG ATC TTG, 5′ rat TNFα GTC GTA GCA AAC CAC CAA G, 3′ rat TNFα GAG CAA TGA CTC CAA AGT AG, 5′ rat IL-6 GGA GTT CCG TTT CTA CCT G, and 3′ rat and IL-6 GTC CTT AGC CAC TCC TTC TG. Primer sequences for PAI-1, RANTES, and TGFβ were described previously [18, 45]. The relative amount of the specific mRNA was normalized to the housekeeping gene β-actin.

### Analysis of data

#### General approach

One-way analysis of variance, followed by the Newman-Keuls test, was used to compare groups. Statistical significance was defined as *p* < 0.05. Data are given as group means ± SEM. SigmaStat 3.5 (Systat Software) was used for statistical analysis.

#### In vivo experiments

Integrated RSNA was recorded as microvolts × seconds (μV s), while individual values were corrected for background noise. Baseline values of RSNA (μV s), mean arterial pressure (mmHg), and heart rate (bpm) were averaged from 10-min periods before intervention.

#### Ex vivo procedures

Intraglomerular ED-1-positive cells were counted in all glomeruli of a given kidney section (120–300 glomeruli, no selection) and expressed as cells per glomerular section. Interstitial PCNA or ED-1-positive cells were counted in 20 medium-power (magnification 250×) cortical views per section and expressed as cells/mm^2^.

Glomerular collagen IV staining was measured by Metaview (Visitron Systems, Puchheim, Germany) in every third glomerulus per cross section, and the stained area was expressed as percentage of the total area of the glomerular tuft.

## Results

### Baseline parameters in vivo

Analysis of MAP, HR, and RSNA did not show differences between groups. Averaged over all groups, MAP was 105 ± 4 mmHg and HR was 377 ± 21 bpm. Amplified, filtered, and noise-corrected mean baseline RSNA was 63.4 ± 6.1 mV s.

### SP involvement in renal innervation

**Fig. 1:**
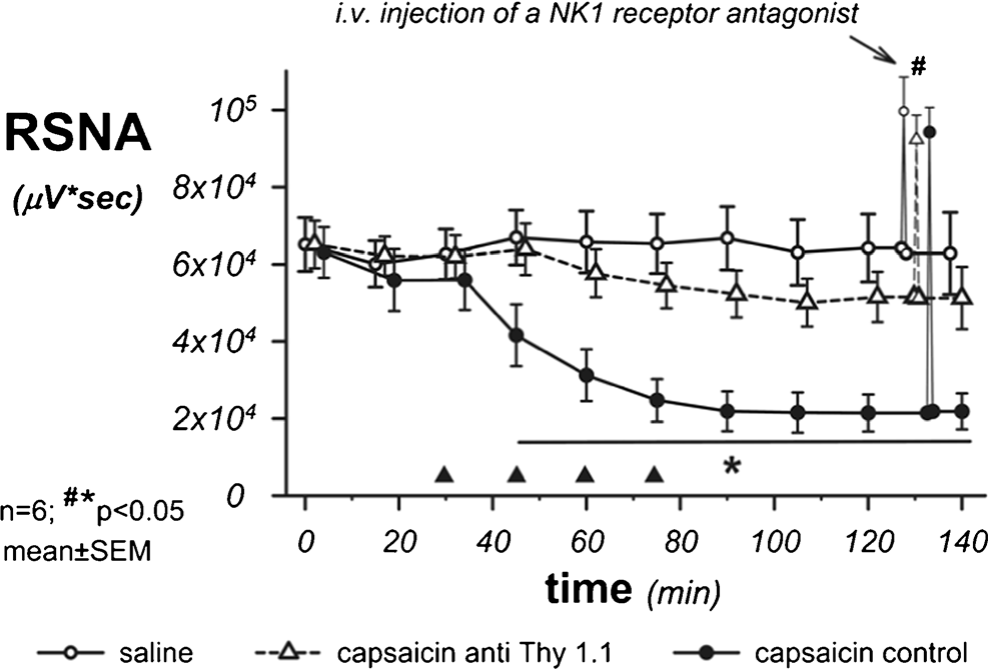
Decreases of renal sympathetic nerve activity after four administrations of a 10 μl bolus of capsaicin or saline into the kidney (filled circles—healthy animals; open triangle—rats with anti-Thy1.1 nephritis; open circle—control group injected with saline). In healthy rats, the capsaicin administration resulted in a steep decrease in sympathetic nerve activity, which was highly significantly reduced in animals with nephritis. Intrarenal saline administration had no effect. Intravenous administration of the substance P receptor antagonist RP67580 (10 mg/kg) was followed by short steep increases in sympathetic nerve activity above baseline levels (duration less than 1 min) in nephritic rats as well as in control animals suggesting SP release from afferent renal nerve fibers upon intrarenal TRPV1 receptors by capsaicin. Interestingly, baseline activity also increased significantly in saline-treated rats after administration of RP67580. This indicates that a tonic SP-dependent sympathoinhibition must also be effective under resting conditions (*n* = 6; ^#^*p < 0.05 versus baseline)

Intrarenal TRPV1-agonism by four consecutive bolus doses of capsaicin into the renal artery induced a sustained RSNA suppression that was severely impaired in rats suffering from experimental nephritis. Intrarenal injections of saline in control animals had no effects (Fig. 1). These results support and extend previous results [13].

During the RSNA suppression, in all respective groups, inhibition of putative SP effects by systemic application of the NK1-receptor (NK-1R) antagonist RP67580 to block the physiological binding site of SP was able to unmask the ensuing RSNA suppression. Other parameters (BP, HR, respiratory rate) were unaffected by either intrarenal TRPV1 receptor stimulation with capsaicin or systemic NK1-antagonism with the chosen dose of RP67580.

Furthermore, inhibition of SP effects by NK1-R blockade increased RSNA even under baseline conditions, when rats were either treated with four bolus injections of saline instead of capsaicin (Fig. 1; see also supplementary material: section A) or no intrarenal TRPV1 stimulation with capsaicin had occurred at all, thus again supporting earlier data of ours [13].

If the NK1 receptor inhibitor RP67580 was injected directly into the kidney, no responses of RSNA or cardiovascular parameters occurred.

### SP and its target receptors NK-1R in the kidney

**Fig. 2:**
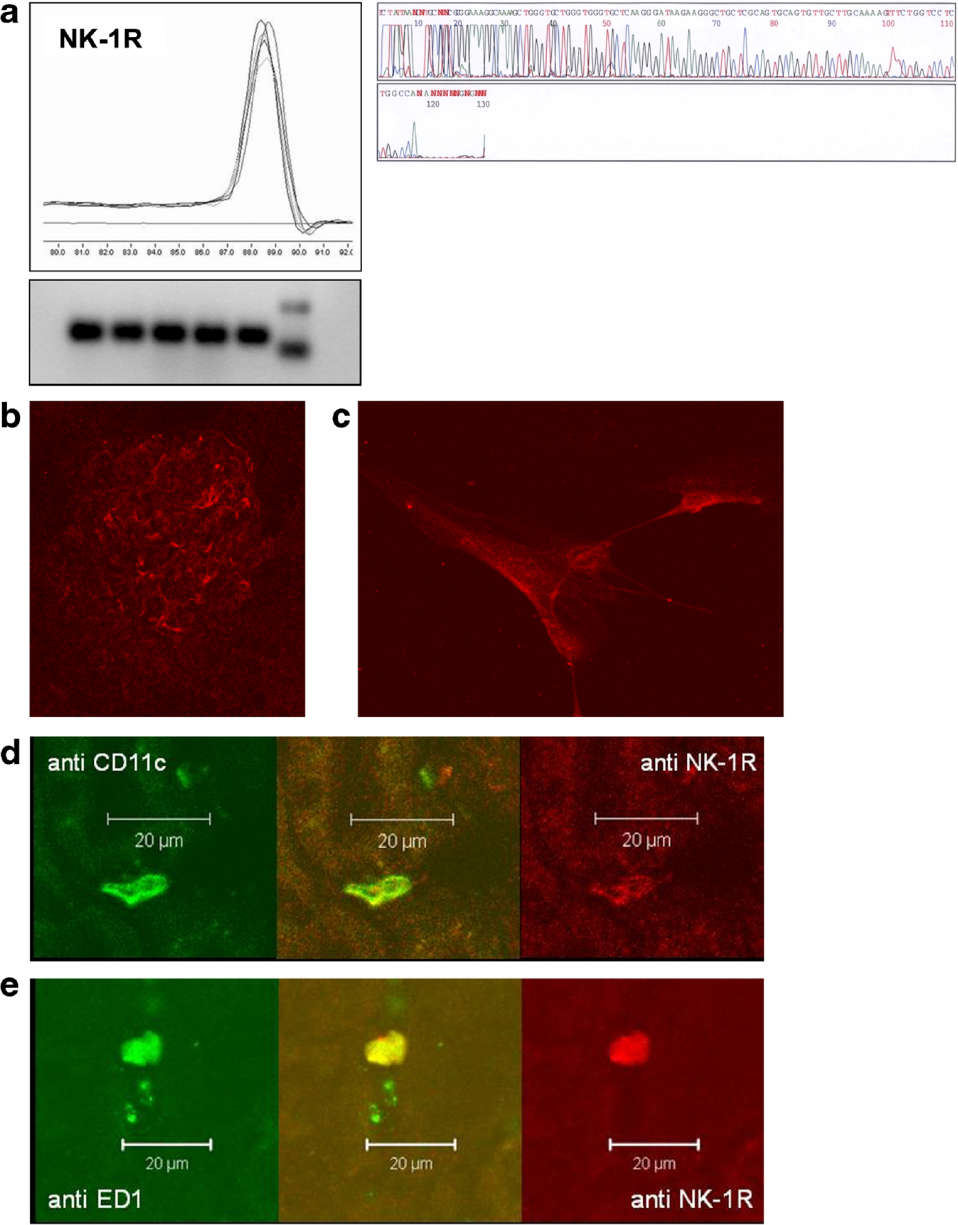
**a** Renal cortical NK-1R mRNA expression of untreated rats was detected by real-time RT-PCR. Amplified PCR products of NK-1R showed identical and specific melting curves as well as an identical and expected length of 125 base pairs in agarose gel electrophoresis. Subsequent sequence analysis revealed a 97% congruence to the published mRNA rat NK-1R transcript. Immunofluorescence analysis by confocal laser scanning microscopy showed NK-1R (red) in a glomerulus (**b**), on cultured mesangial cells (**c**), on ED1-positive macrophages (green; **d**), and on CD11c-positive dendritic cells (green; **e**). The yellow merge indicates colocalization of NK-1R with ED1-positive cells and CD11c-positive dendritic cells, respectively

The NK-1-receptor was detected by RT-PCR in rat renal cortex. The specificity of amplified products was confirmed by melting curve analysis and gel electrophoresis. The PCR products (*n* = 5) showed specific melting curves as well as an identical and expected length of 125 base pairs in subsequent agarose gel electrophoresis (Fig. 2a). As 108 out of 108 readable bases matched the NK-1R by 97%, congruence between the NK-1R coding mRNA in rat kidney and the published mRNA for the rat NK-1R transcript (GeneBank accession no. NM012667.1) can be expected.

In order to analyze the cellular distribution of the NK-1R in renal cortex, cryostat sections of cortical tissue as well as mesangial cell cultures were investigated using immunofluorescence staining and confocal laser scanning microscopy. We found that the receptor is expressed within glomeruli (Fig. 2b) and by mesangial cells (Fig. 2c). Using double immunofluorescence staining of NK-1R together with cell-specific markers, we observed that also interstitial ED1-positive macrophages as well as interstitial CD11c-positive DCs express the receptor (Fig. 2 d and e).

**Fig. 3:**
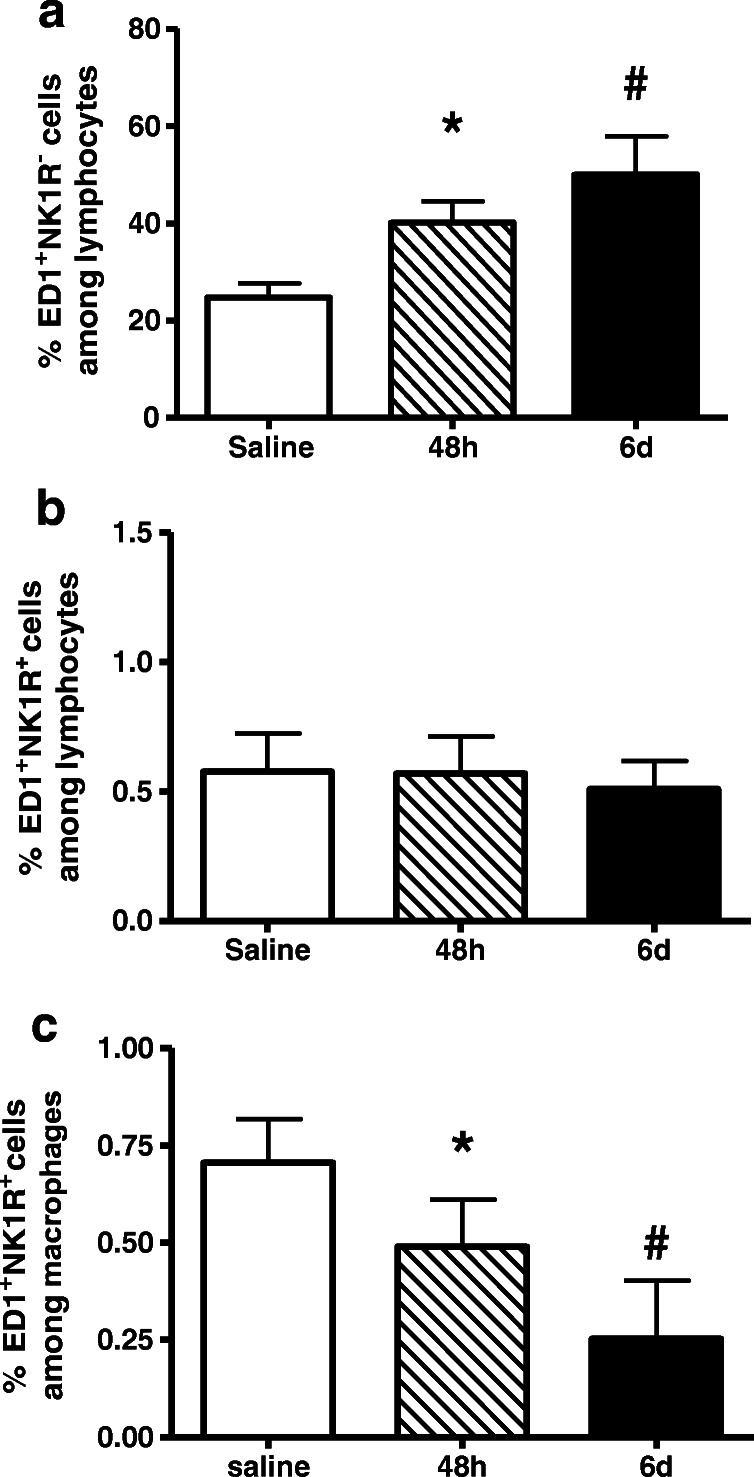
NK1-R expression on macrophages in glomerulonephritis. Rats with anti-Thy1.1-induced glomerulonephritis (filled bars) or with saline (stripped bar) for control group. Frequency of macrophages (**a**), ED1^+^NK1R^+^ cells among lymphocytes (**b**) as well as ED1^+^NK1R^+^ cells among macrophages (**c**), and the mean fluorescence intensity of NK-1R expression on macrophages (**d**) were detected by FASC analysis. Data are given as mean ± SEM; *n* = 6, *p < 0.05 versus saline; ^#^*p* < 0.05 versus saline

Renal leukocytes were isolated from animals 2 and 6 days after nephritis induction and investigated by FACS analysis. The frequency of ED1-positive macrophages was increased at both time points (Fig. 3a; see also supplementary material: section C); however, only a smaller percentage of macrophages expressed NK1R, and the count of ED1^+^NK1R^+^ cells among lymphocytes was unaltered compared with the control group (Fig. 3b). Focusing on the frequency of ED1^+^NK1R^+^ cells among macrophages, a decrease of the cell number could be measured on day 6 compared with saline control (Fig. 3c). In addition, the mean fluorescence intensity of NK1R expression on macrophages was analyzed. No changes in an upregulation of NK1R on each single cell could be detected at the indicated time points in nephritis animals compared with control animals.

**Fig. 4:**
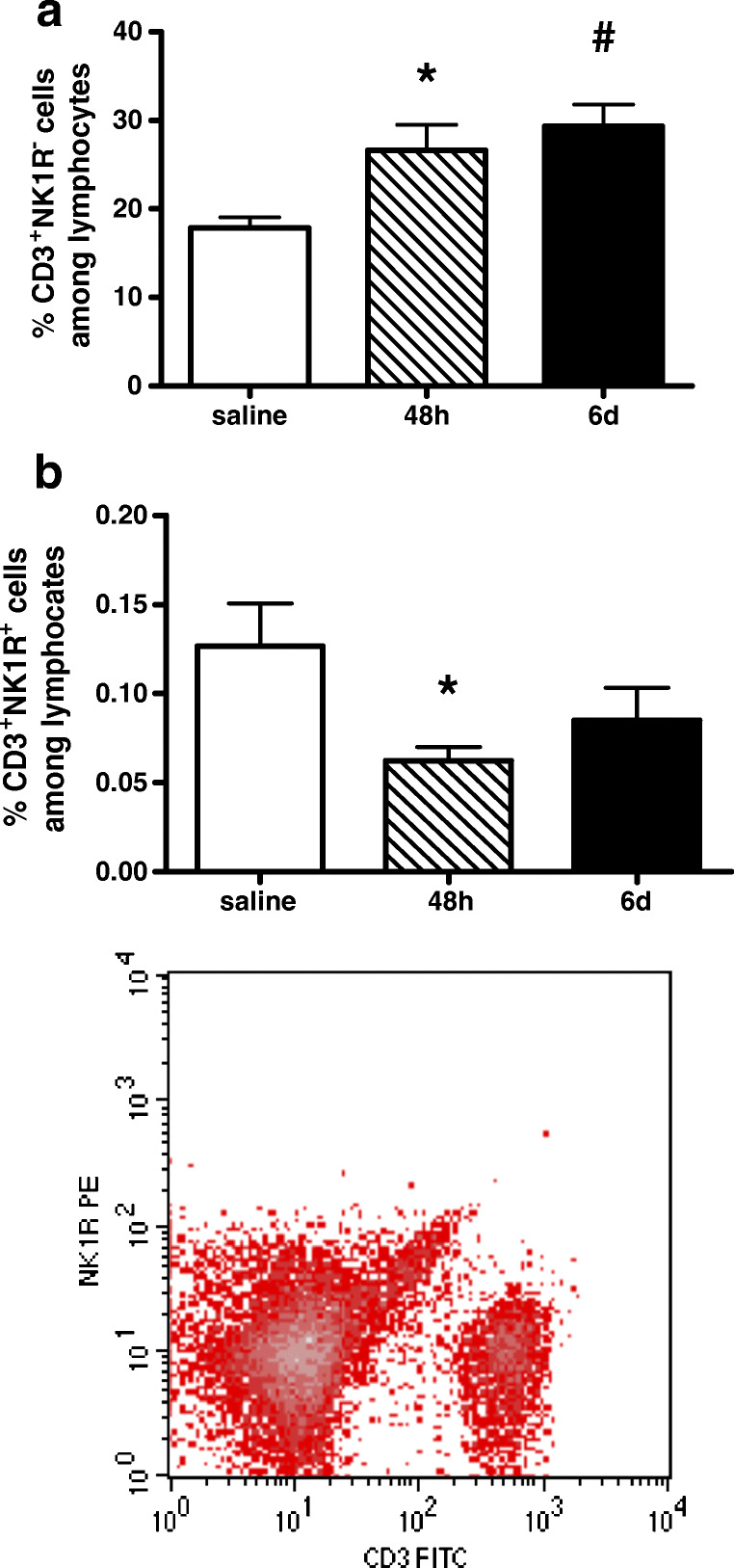
NK1-R expression on T cells in glomerulonephritis. Rats with anti-Thy1.1-induced antibody glomerulonephritis (filled bars) or with saline (stripped bar) for control group. NK1-R expression was measured by FACS analysis. Although the frequency of T cells is increased (**a**), only a small percentage of CD3-positive T cells express NK-1R (**b**). Data are given as mean ± SEM; *n* = 6, *p < 0.05 versus saline; ^#^*p* < 0.05 versus saline

Furthermore, CD3-positive T cells were investigated with respect to expression and regulation of NK1R during nephritis. As shown in Fig. 4a, CD3-positive cells were increased in anti-Thy1.1 nephritis. Only a small percentage of T cells expressed NK-1R, and the frequency of CD3^+^NK1R^+^ cells was significantly decreased 48 h after nephritis induction (Fig. 4b).

**Fig. 5:**
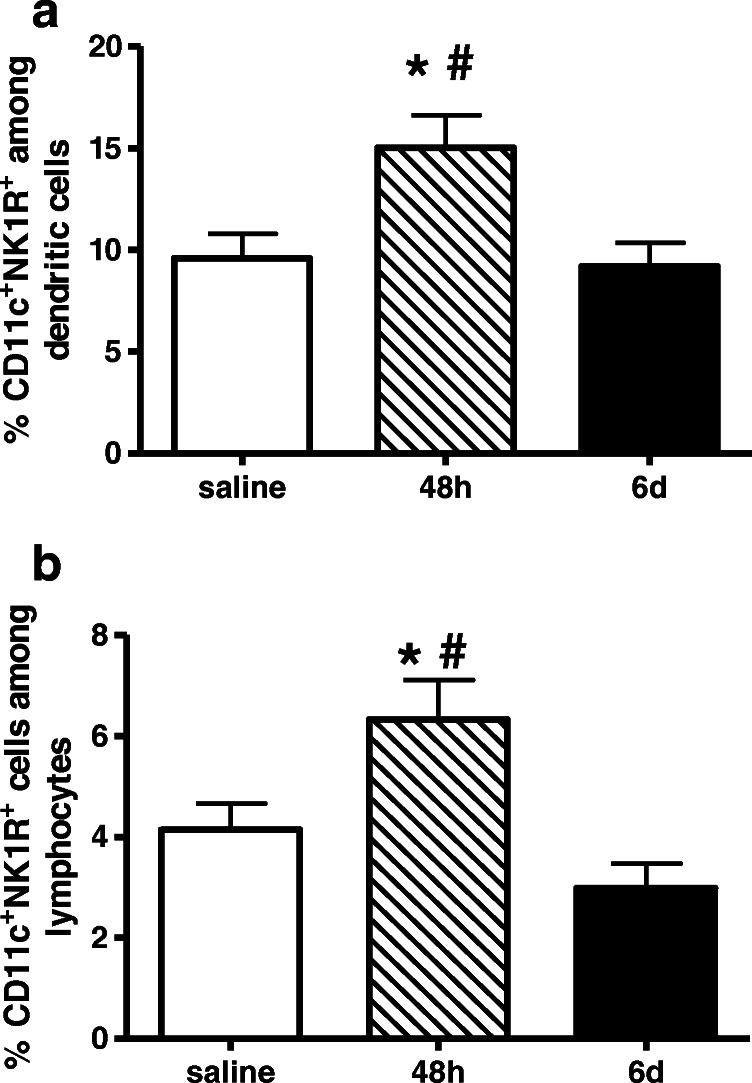
NK1-R expression on dendritic cells in glomerulonephritis. Rats with anti-Thy1.1 antibody induced glomerulonephritis (filled bars) or with saline (stripped bar) as control group. NK1-R expression was measured by FACS analysis. Frequency of NK-1R^+^CD11c^+^ among lymphocytes (**a**) as well as among dendritic cells (**b**) was increased 48 h after antibody induction (**b**). Data are given as mean ± SEM; *p < 0.05 versus saline; ^#^*p* < 0.05 versus 6 days (6d)

Interestingly, NK1-R is mainly expressed on CD11c-positive DCs (Fig. 5a). The percentage of CD11c^+^NK1R^+^ among DCs was increased 2 days after anti-Thy1.1 antibody injection (Fig. 5b), whereas on day 6, the count of CD11c^+^NK1R^+^ cells was restored in the inflamed kidney.

### SP—functional role in experimental nephritis

To further clarify the question whether afferent nerve fibers from the kidney and its mediators influence renal inflammation, NK1-receptors, the receptors of SP, were blocked with the specific NK1-R antagonist aprepitant. Antagonism of SP led to a significant reduction in proteinuria and albuminuria as shown in Table 1 on day 6 after nephritis induction.

**Table 1:** Urinary albumin and protein excretion in anti-Thy 1.1 nephritis and controls (time after induction of nephritis; * *p* < 0.05 anti-Thy1.1 plus aprepitant versus anti-Thy1.1; *n* = 6)

		24 h	48 h	6 days
Albuminuria	Anti-Thy 1.1 plus aprepitant	3.6 ± 1.3 mg/24 h	8.5 + 2.4 mg/24 h	5.3 + 1.7 mg/24 h*
	Anti-Thy 1.1	4.3 ± 0.8 mg/24 h	8.0 ± 1.1 mg/24 h	19.8 ± 5.2 mg/24 h
	Saline plus aprepitant	0.2 ± 0.1 mg/24 h	0.2 ± 0.08 mg/24 h	0.2 ± 0.07 mg/24 h
Proteinuria	Anti-Thy 1.1 plus aprepitant	6.4 ± 1.2 mg/24 h	18.1 + 4.6 mg/24 h*	12.7 + 1.4 mg/24 h*
	Anti-Thy 1.1	7.4 ± 1.8 mg/24 h	33.5 ± 14.1 mg/24 h	78.7 ± 15.8 mg/24 h
	Saline plus aprepitant	4.8 ± 0.6 mg/24 h	5.2 ± 0.8 mg/24 h	4.9 ± 0.7 mg/24 h

**Fig. 6:**
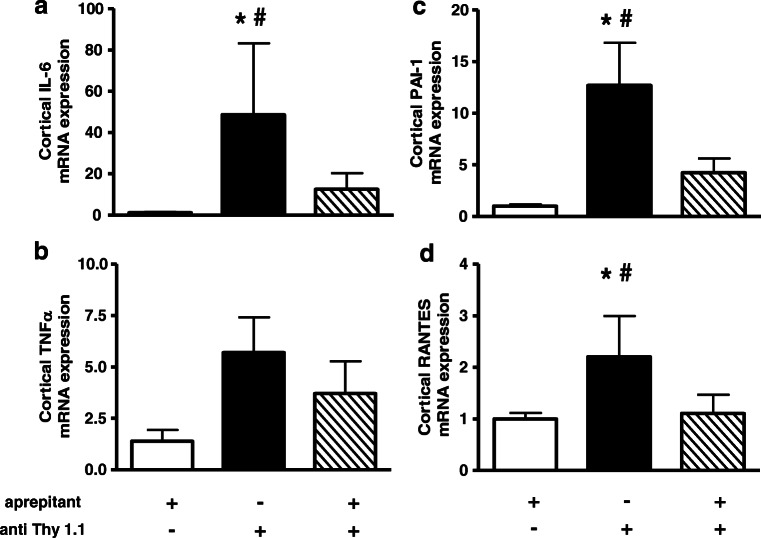
Aprepitant exerts an anti-inflammatory effect in anti-Thy1.1 nephritis. Rats were pretreated with 5 mg/kg aprepitant i.p. 12 h and 1 h before injection of anti-Thy1.1 mAb and every 24 h after antibody challenge (diagonal bars). Further animals received vehicle plus anti-Thy1.1 (black bars) or aprepitant plus saline instead of anti-Thy1.1 mAb (white bars). Renal cortical mRNA expression of TNFα, PAI-1, IL-6, and RANTES was detected by real-time RT-PCR. The relative amount of mRNA was normalized against the housekeeping gene β-actin. Data are given as mean ± SEM; *p < 0.05 versus aprepitant plus vehicle; ^#^*p* < 0.05 versus anti Thy1.1 mAb plus aprepitant

Renal inflammation was examined by determination of pro-inflammatory cytokines and markers of sclerosis. Pretreatment with aprepitant attenuated renal cortical mRNA expression of the pro-inflammatory cytokines IL-6 and TNF-α as well as expression of PAI-1 and RANTES (CCL5), which is known as early marker of sclerosis (Fig. 6a–d; see also supplementary material: section D).

**Fig. 7:**
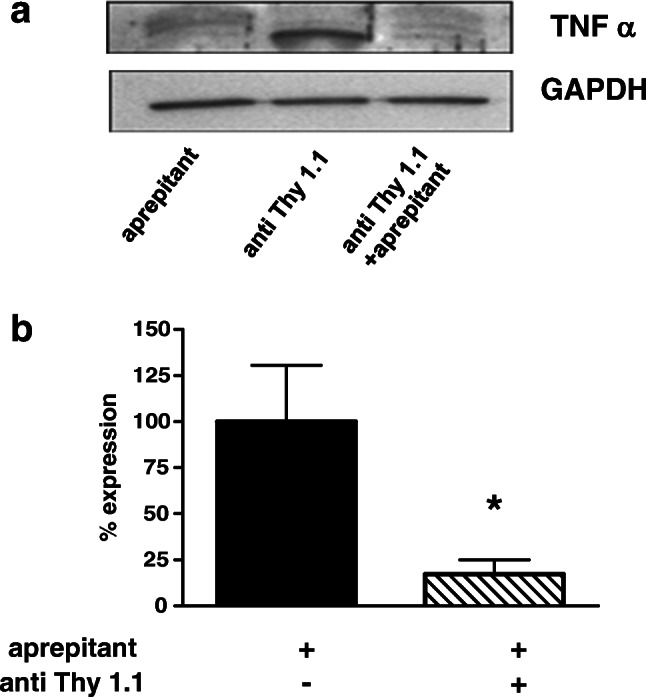
NK-1R blockade decreases protein synthesis of TNFα. Rats were pretreated with 5 mg/kg aprepitant i.p. 12 h and 1 h before injection of anti-Thy1.1 mAb and every 24 h after antibody challenge. TNFα protein was detected by Western Blot and GAPDH was used as house keeping gene to ensure equally protein amounts. The small western blot shows pooled lysates of renal cortical tissues of all three groups (**a**). **b** Saline plus anti-Thy-1.1 mAb and aprepitant plus anti-Thy1.1 mAb were quantified. Data are given as mean ± SEM; *n* = 6; *p < 0.05

Western blot analysis of renal cortical tissue disclosed a significant inhibition of protein synthesis of TNF-α, a key player in inflammatory processes. As shown in Fig. 7 a and b, lower amounts of protein were measured in anti-Thy1.1 nephritis rats pretreated with NK1-R antagonist compared with non-pretreated animals. Pooled lysates demonstrated an increase of TNFα protein amount in nephritis rats compared with the control group.

**Fig. 8:**
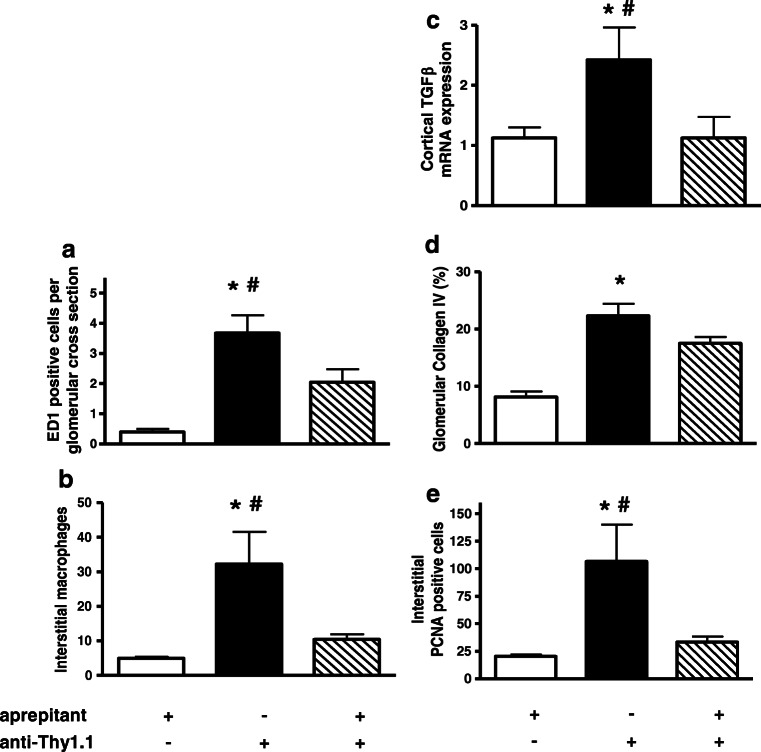
Aprepitant pretreatment reduced renal macrophages (**a** and **b**), TGFβ expression (**c**), deposition of collagen IV (**d**), and interstitial proliferation (**e**) anti-Thy1.1 nephritis. Rats were pretreated with 5 mg/kg aprepitant i.p. 12 h and 1 h before injection of anti-Thy1.1 mAb and every 24 h after antibody challenge (diagonal bars). Control animals received either vehicle plus anti-Thy1.1 mAb (black bars) or aprepitant plus saline instead of anti-Thy1.1 mAb (white bars). Alterations of macrophages were determinated by immunohistochemistry. Renal cortical mRNA expression of TGFβ was detected by real-time RT-PCR. The relative amount of mRNA was normalized against the housekeeping gene β-actin. Data are given as mean ± SEM; *n* = 6, *p < 0.05 versus aprepitant plus vehicle; ^#^*p* < 0.05 versus anti Thy 1.1 mAb plus aprepitant

In addition, changes of renal macrophage counts were determined. Both glomerular and interstitial ED1-positive macrophages were increased in anti-Thy1.1 nephritis animals compared with control rats. Pretreatment with aprepitant significantly reduced acceleration of macrophage infiltration into glomeruli as well as into renal interstitium confirming the anti-inflammatory effect of NK1-R blockade (Fig. 8 a and b).

Furthermore, alterations of renal fibrosis markers were examined. Induction of nephritis led to a significant deposition of collagen IV accompanied with an increase of renal TGFβ mRNA expression. Pretreatment with NK1-R antagonist aprepitant reduced renal TGFβ mRNA expression as well as collagen IV deposition in glomeruli (Fig. 8 c and d).

In addition to fibrosis markers, cell proliferation was investigated by quantification of interstitial PCNA-positive cells. Induction of anti-Thy-1.1 nephritis led to a significant increase of proliferating cells and antagonism of NK1-R with aprepitant restored interstitial proliferation (Fig. 8e).

## Discussion

We demonstrate for the first time that substance P (SP) released from afferent peptidergic neural fibers promoted inflammatory processes in experimental nephritis in a complex manner. Recordings of efferent renal sympathetic nerve activity showed that the SP-dependent sympathoinhibitory reno-renal reflex pathway [13] was impaired. Furthermore, dendritic cells expressing a SP receptor were increased in nephritis, and pharmacological blockade of this receptor alleviated the inflammatory damage.

Our data again suggest that SP released from afferent nerve fibers is involved in a neurogenic-paracrine mechanism of sympathoinhibition of long duration, which already tones down sympathetic activity under baseline conditions. This sympathoinhibitory mechanism could be shown to be seriously impaired in experimental nephritis, a regional disease situation without major systemic consequences.

Furthermore, mainly DCs expressed an increased number of NK1-receptors during the initial phase of the experimental glomerulonephritis in rats. Since the inhibition of NK1-R in anti-Thy1.1 nephritis, whose agonist SP is released from renal peptidergic afferent nerve fibers, ameliorated significantly the inflammatory and structural damage in the course of the disease, our findings demonstrate the important pathogenic role of intrarenal DCs in response to neurogenically released SP in this experimental model of mesangioproliferative glomerulonephritis.

### SP involvement in renal innervation

We could again induce SP-dependent long-lasting sympathoinhibition by direct intrarenal administration of a TRPV1 receptor agonist, capsaicin [13], which was significantly impaired in nephritis. This sympathetic reflex mechanism could not be triggered when a comparable amount of capsaicin was administered intravenously. It must therefore originate within the kidney so that it can be mediated via renal afferent nerve fibers [59]. An SP receptor blocker was able to antagonize the long-lasting sympathetic inhibition seen after intra-arterial administration of capsaicin into the kidney. Therefore, this sympathetic inhibition must be dependent on SP. Since the administration of a SP antagonist already led to an increase in sympathetic activity in control animals, whose intrarenal TRPV1 receptors were not stimulated by capsaicin, these SP-dependent neurogenic mechanisms of sympathoinhibition obviously subserve tonically sympathoinhibitory afferent pathways.

How SP from afferent nerve fibers could affect sympathetic nerve tracts has not yet been clarified. A close relationship of SP-positive nerve fibers to afferent and efferent arterioles and glomeruli was already described more than three decades ago [47]. We could confirm this finding with respect to SP [13, 48]. However, such an interaction must not necessarily occur in the kidney since peptides like SP could be secreted along the whole surface of the axon and not only at the nerve endings [58]. Neurons with afferents from the kidney are located in the dorsal root ganglia in the vicinity of sympathetic fibers. Likewise, sympathetic neurons and afferent nerve fibers occur together in the aorticorenal ganglion, from which the final sympathetic pathways to the kidney originate [13].

We now report a severe impairment of SP-dependent sympathetic depression during experimental nephritis, which according to our further data should be characterized by an increased neurogenic turnover and secretion of SP. To what extent an increased secretion of SP in the kidney can impair mechanisms of afferent renal innervation requires further specialized experiments that should also address the consequences of putatively antidromal depolarization (e.g. towards the periphery and not the CNS) of afferent renal nerve fibers during increased SP release in inflammation [8].

### SP and its target receptors NK-1R in the kidney

Renal denervation reduced concentrations of SP in the respective kidneys to hardly detectable levels 7 days after the procedure pointing to neurogenic sources of SP [48]. Interestingly, the cellular distribution of the primary receptor for SP in the kidney has so far not raised too much attention. In our experiments, this receptor, NK1-R, was detected on mesangial cells in glomeruli as well as on macrophages and DCs in the renal interstitium.

As proven by very many publications, neuropeptide SP released from nerve endings of primary neurons is well known to exert pro-inflammatory effects via activation of NK1-R at the site of an inflammation. It has been shown that SP enhanced lymphocyte proliferation and induced the release of inflammatory mediators and thereby promoting inflammation in a wide variety of organs and conditions like in the liver, in the joints, in the respiratory tract, and in the skin [15, 40, 43, 55]. Macrophages play an important role in the induction of renal injury and are well known for their potency to secrete cytokines such as TNFα, IL-1, and IL-6 after SP stimulation [43]. In our experiments, NK1-R were predominantly present on the surface of DCs. It has been known for a long time that murine and human DCs express NK1-R [37]. Moreover, we could demonstrate a close vicinity of DCs to primary sensory neurons in the kidney [58]. Eventually, the frequency of NK1-R^+^CD11c^+^ cells was enhanced 48 h after nephritis induction suggesting a modulatory function of DCs in the development of kidney inflammation. A possible implication might be that DCs mediate T cell proliferation via NK1-R binding. Indeed, it has been described that SP induces activation of nuclear factor-κB in general [41] and of note in murine DC [38]. This transcription factor regulates expression of MHCII, co-stimulatory molecules (CD86), induces cytokines like IL-12 as well as TNFα in DCs, and therefore plays a pivotal role in the regulation of antigen presentation and the initiation of T cell–dependent immune responses [17, 44, 61].

### SP—functional role in experimental nephritis

Pretreatment with the selective NK1-R antagonist aprepitant in anti-Thy1.1 nephritis revealed a strong anti-inflammatory effect of SP blockade. This effect was detectable by significant reduction of physiological, proliferative, and pro-inflammatory parameters.

PAI-1 overexpression has been associated with several acute and chronic inflammatory disorders [35]. In case of renal inflammation, PAI-1 has been shown to mediate inflammatory cell infiltration and its pathophysiological consequences in crescentic glomerulonephritis [28] as well as in anti-Thy1.1 nephritis [21]. Being involved in a lot of inflammatory diseases, IL-6 [42] and RANTES [33] obviously exhibit their pro-inflammatory and chemoattractant potential also in renal disease [52, 60].

In contrast to TNFα protein production, no significant decrease in cortical TNFα mRNA expression was measured at the end of the experiment. This might implicate that TNFα gene expression was recovering whereas protein level was still down-modulated. In a mice model of nephrotoxic nephritis, the group of Khan et al. demonstrated the central role of TNFα in the beginning and the progression of nephritis. Blockade of TNFα with a monoclonal antibody reduced renal inflammation and maintained renal function [27]. Hence, inhibition of TNFα synthesis might lead to an abrogation of its pro-inflammatory effects such as secretion of other pro-inflammatory cytokines, chemokines, and growth factors as well as recruitment of inflammatory immune cells at the side of inflammation. Indeed, challenge of NK1-R antagonist decreased both cortical IL-6 and RANTES mRNA expression, and a significant reduction of ED1-positive macrophages within the glomeruli and the renal interstitium was observed.

Since inflammation and the secretion of cytokines seem to be a prerequisite for fibrosis and the ensuing structural lesions [6, 46], we also observed a strong inhibition of TGFβ expression and [7] decrease of PCNA-positive cells.

Further specialized experiments must prove in how far SP inhibition could be used as therapeutic means to alleviate the deleterious effects of SP-dependent neurogenic mechanism on renal disease not only in mere inflammation but perhaps also in arterial hypertension.

K. Amann and R. Veelken were supported by a grant-in-aid from the Deutsche Forschungsgemeinschaft (AM 93/10-1; VE 104/4-1) and Interdisciplinary Center for Clinical Research (IZKF) of the University Erlangen.

**Table:** 

	Conceived of or designed study	Performed research	Analyzed data	Contributed new methods or models	Wrote the paper
Kristina Rodionova	x	x	x		x
Karl F. Hilgers			x	x	
Eva-Maria Paulus		x	x		
Gisa Tiegs	x		x	x	
Christian Ott			x		
Roland Schmieder			x		
Mario Schiffer				x	
Kerstin Amann	x		x	x	
Roland Veelken	x	x	x	X	x
Tilmann Ditting	x	x	x	X	x

## Electronic supplementary material

ESM 1 (DOCX 15685 kb)
