# Supplementary material for: Neurogenic tachykinin mechanisms in experimental nephritis of rats
Source: Pflugers Arch. 2020 Oct 17;472(12):1705–17. doi: 10.1007/s00424-020-02469-z (PMC7691313; doi:10.1007/s00424-020-02469-z)
Supplement: Supplementary file 1 — (DOCX 15685 kb) [file 424_2020_2469_MOESM1_ESM.docx]

***Supplementary Material***

*Revision 1 PAEJ –D-20-00139*

**Neurogenic Tachykinin Mechanisms in Experimental Nephritis of Rats**

Kristina Rodionova^1^, Karl F. Hilgers^1^, Eva-Maria Paulus^1^, Gisa Tiegs^4^, Christian Ott^1,2^, Roland Schmieder^1^, Mario Schiffer^1^, Kerstin Amann^3^, Roland Veelken^1,2*^ ,Tilmann Ditting^1,2*^

^1^ Department of Internal Medicine 4 (Nephrology und Hypertension),

University of Erlangen, Erlangen, Germany

^2^ Department of Internal Medicine 4 (Nephrology und Hypertension),

Paracelsus Private Medical School, Klinikum Nuremberg, Nuremberg, Germany

^3^ Department of Nephropathology, University of Erlangen, Erlangen, Germany

*^4^*Center of Internal Medicine, University Medical Center Hamburg-Eppendorf, Hamburg,

**Section A: Representative Recordings of Renal Sympathetic Nerve (RSNA) Activity and Cardiovascular Parameters**


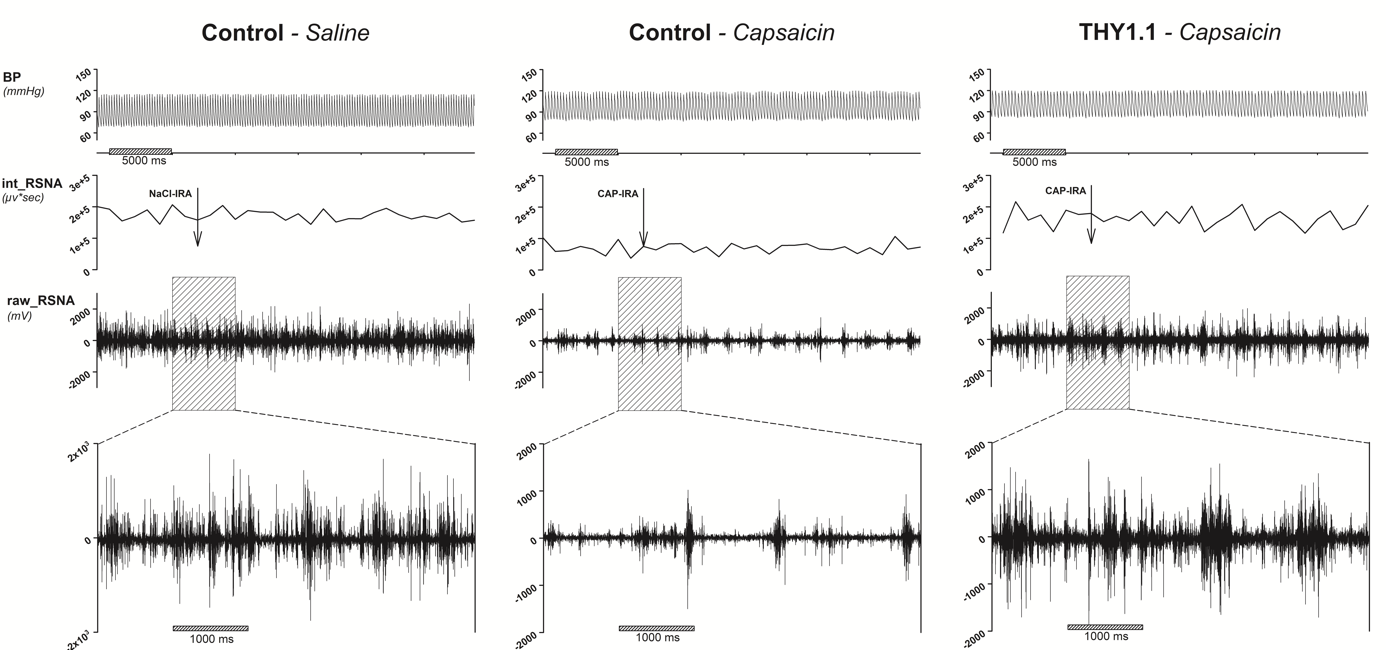


***Fig 1***: Displayed are representative recordings of blood pressure (BP) and efferent renal sympathetic nerve activity (RSNA) at the timepoint of direct injection of either normal saline or capsaicin (1.0 x10^-6^M) into the kidney via the renal artery (IRA), which was the 4^th^ IRA injection in our experimental protocol (also see Figure 1 in the main article). The Left panel shows a recording of a healthy control which was IRA injected with normal saline, the middle panel shows a recording from a healthy control which was injected for the 4^th^ time with increasing doses of capsaicin, and the right panel shows a recording from a rat with anti Thy1.1 nephritis. Capsaicin injection into one kidney did not induce any reproducible momentary response of contralateral RSNA, but it rather induced a long lasting RSNA suppression over the time course of the experimental protocol, as previously described (Ditting et al, Hypertension, 2012). Saline injection did not induce any change in RSNA. However, in rats with Thy1.1 nephritis this long-lasting RSNA suppression was significantly impaired. (BP = pulsatile blood pressure; int_RSNA = RSNA integrated over 1 second intervals; raw_RSNA = RSNA raw neurogram; RSNA data displayed here are not corrected for noise).


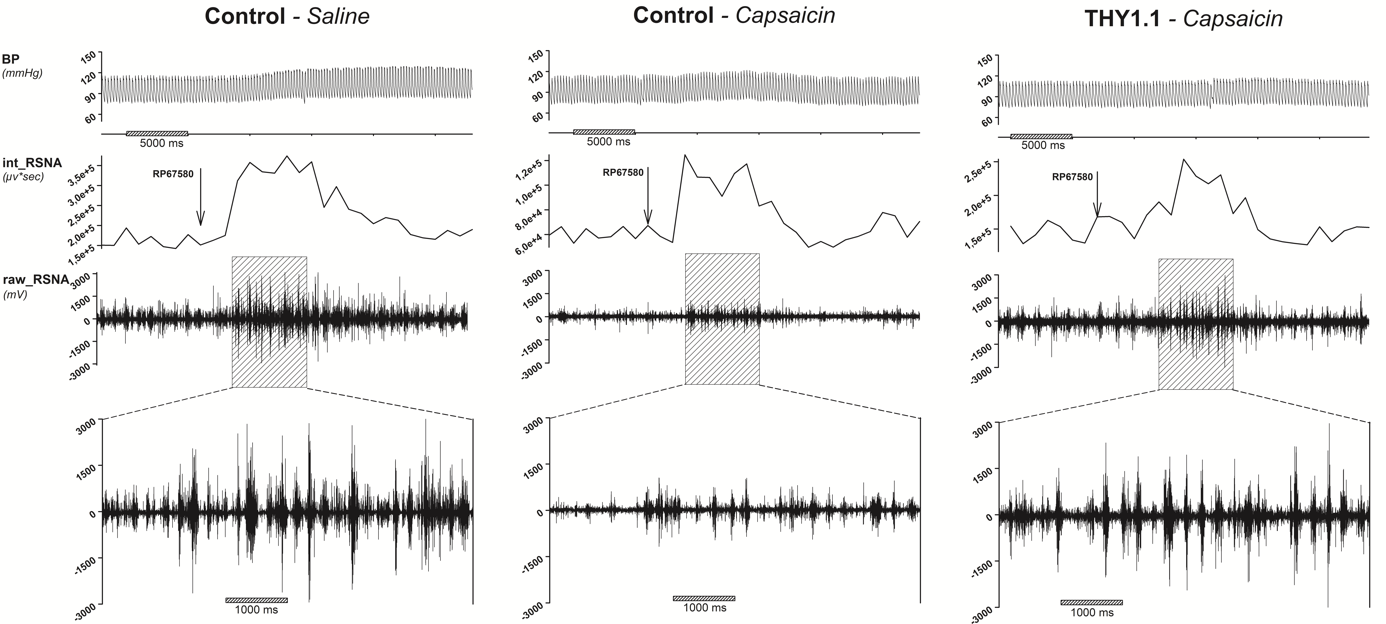


***Fig 2:*** Displayed are representative recordings of BP and RSNA at the timepoint in the experimental protocol, when the NK1-receptor-antagonist RP67580 was injected i.v. to unmask RSNA after the capsaicin induced long-lasting suppression. The Left panel shows a recording of a healthy control which was IRA injected with normal saline before, thus there was no suppression of baseline RSNA but still a steep but short-lived increase of RSNA. The middle panel shows a recording from a healthy control which was IRA injected with increasing doses of capsaicin which induced a subtotal RSNA suppression, being unmasked by RP67580 given intravenously. The right panel shows a recording from a rat with Thy1.1 nephritis, which did hardly show RSNA suppression after increasing IRA doses of capsaicin. (BP = pulsatile blood pressure; int_RSNA = RSNA integrated over 1 second intervals; raw_RSNA = RSNA raw neurogram; RSNA data displayed here are not corrected for noise; note that int_RSNA data are scaled for data range).

**Section B: Recordings of afferent renal nerve activity**

**
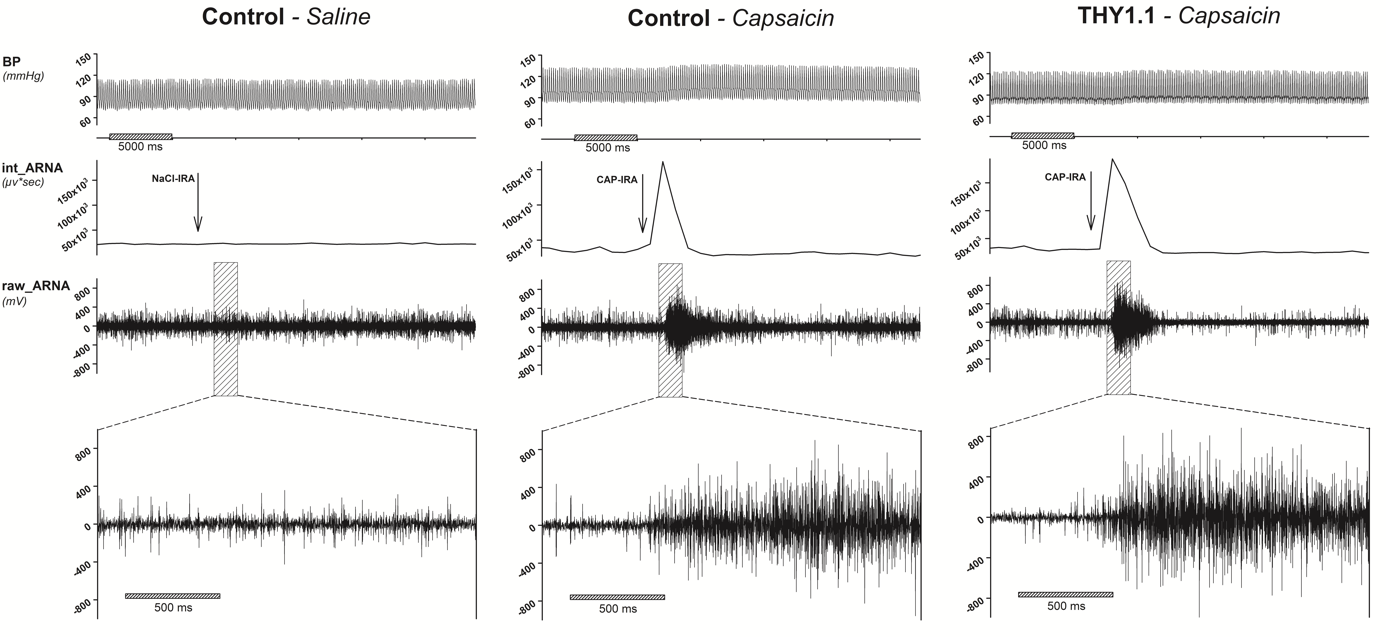
**

***Fig 3:*** Displayed are representative recordings of afferent renal nerve activity (ARNA) at the timepoint of direct injection of either normal saline or capsaicin (1.0 x10^-6^M) into the kidney via the renal artery (ARNA ipsilaterally). The left panel shows a recording of a healthy control which was injected with normal saline instead of capsaicin. The middle panel shows a recording from a healthy control which was injected with capsaicin and the right panel shows a recording from a rat with anti Thy1.1 nephritis. Capsaicin induced similar short-lived increases of ARNA in healthy controls, as previously described (Ditting et al, Hypertension, 2012) and in rats with Thy1.1 nephritis. Saline injection did not induce any ARNA response.

ARNA was recorded from the distal cut end of a left renal nerve. ARNA like RSNA signals were amplified 50,000 times and filtered using a band pass amplifier CyberAmp 320; Axon Instruments, Foster City, CA, USA).The signal was channeled to an A/D oscilloscope (HM 305-3; Hameg, Frankfurt, Germany) and an audio amplifier (AM8 audio monitor; Grass-Telefactor, West Warwick, RI, USA) for visual and auditory evaluation. With an optimal signal achieved the nerve bundle was fixed to the electrode using silicone adhesive (Bisico S4i; Bielefelder Dentalsilicone, Bielefeld, Germany).

**
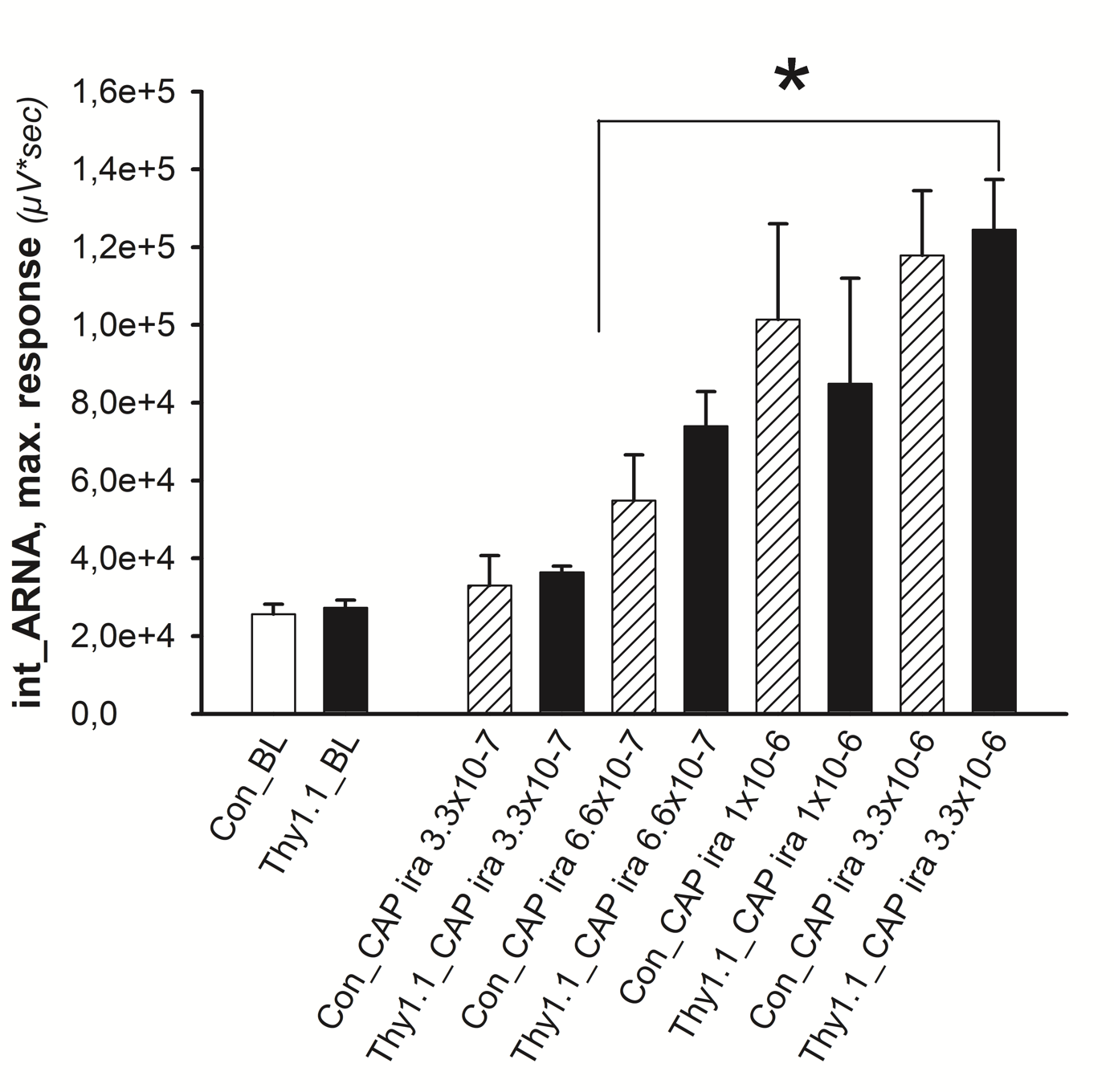
**

***Fig 4:*** Displayed are the ARNA responses due to increasing doses of Capsaicin given via the renal artery (IRA). White bares denote healthy controls, black bars denote rats with Thy1.1 nephritis. BL = baseline value, which did not changed due to IRA injection of normal saline. Increasing doses of IRA capsaicin induced increasing ARNA responses. However, there was no difference between healthy controls and rats with Thy1.1 nephritis. (*p<0.05 CAP ira *vs.* BL; data displayed here are not corrected for noise).

**Section C: Representative images of immunohistochemical analyses**

***ED1 imaging***


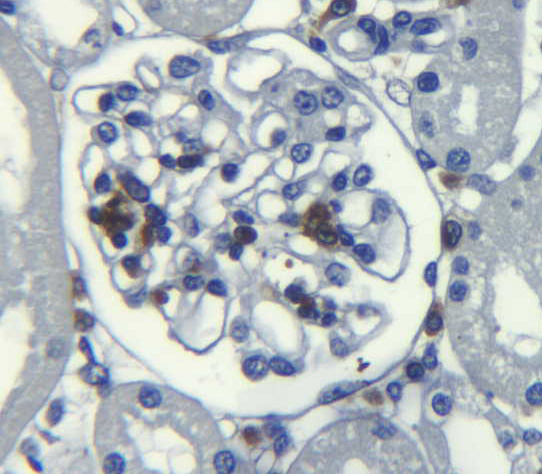


**without aprepitant treatment**


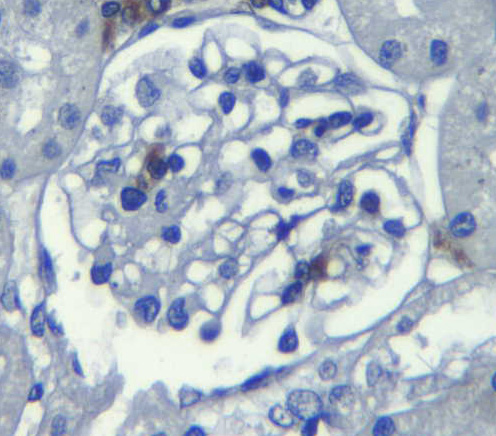


**with aprepitant treatment**

***Fig 5:*** Representative ED 1 staining in rats suffering from mesangioproliferaitve nephritis induced by anti Thy1.1 monoclonal antibodies (upper panel) and nephritic animals pretreated with 5 mg/kg aprepitant i.p. 12h and 1h before injection of anti-Thy1.1 and every 24h after antibody challenge (lower panel). Seven days after the inducting of Thy1.1 nephritis ED 1 staining was significantly reduced in animals pretreated with aprepitant.

***PCNA imaging***


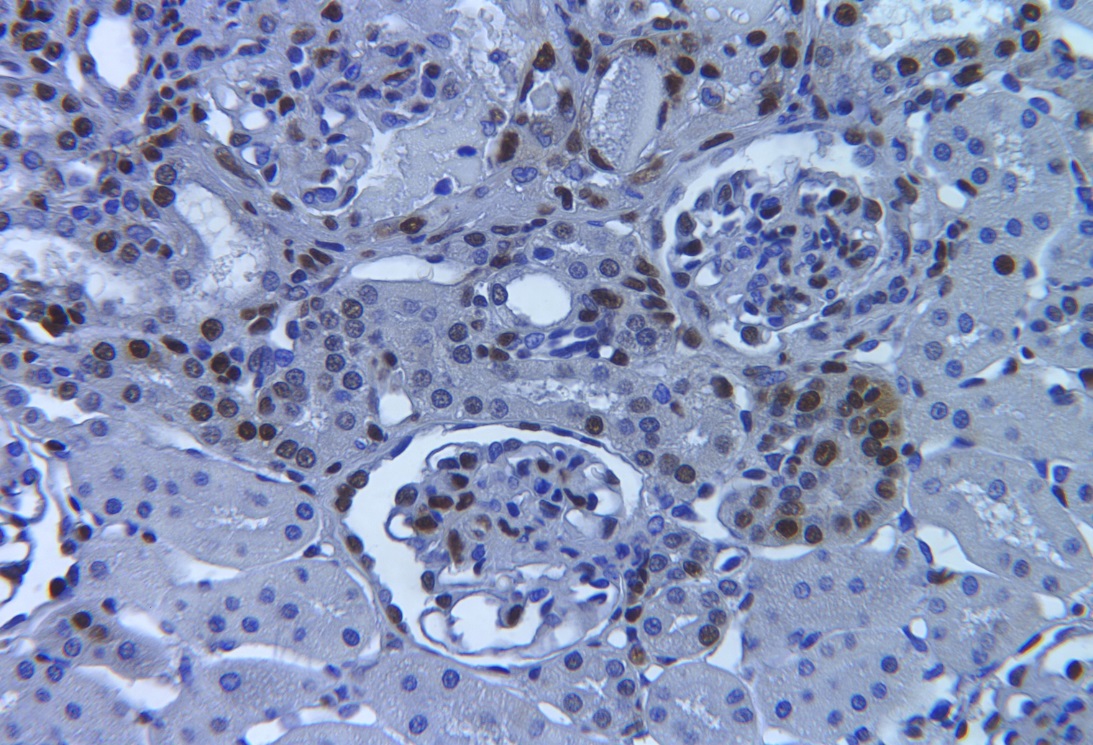


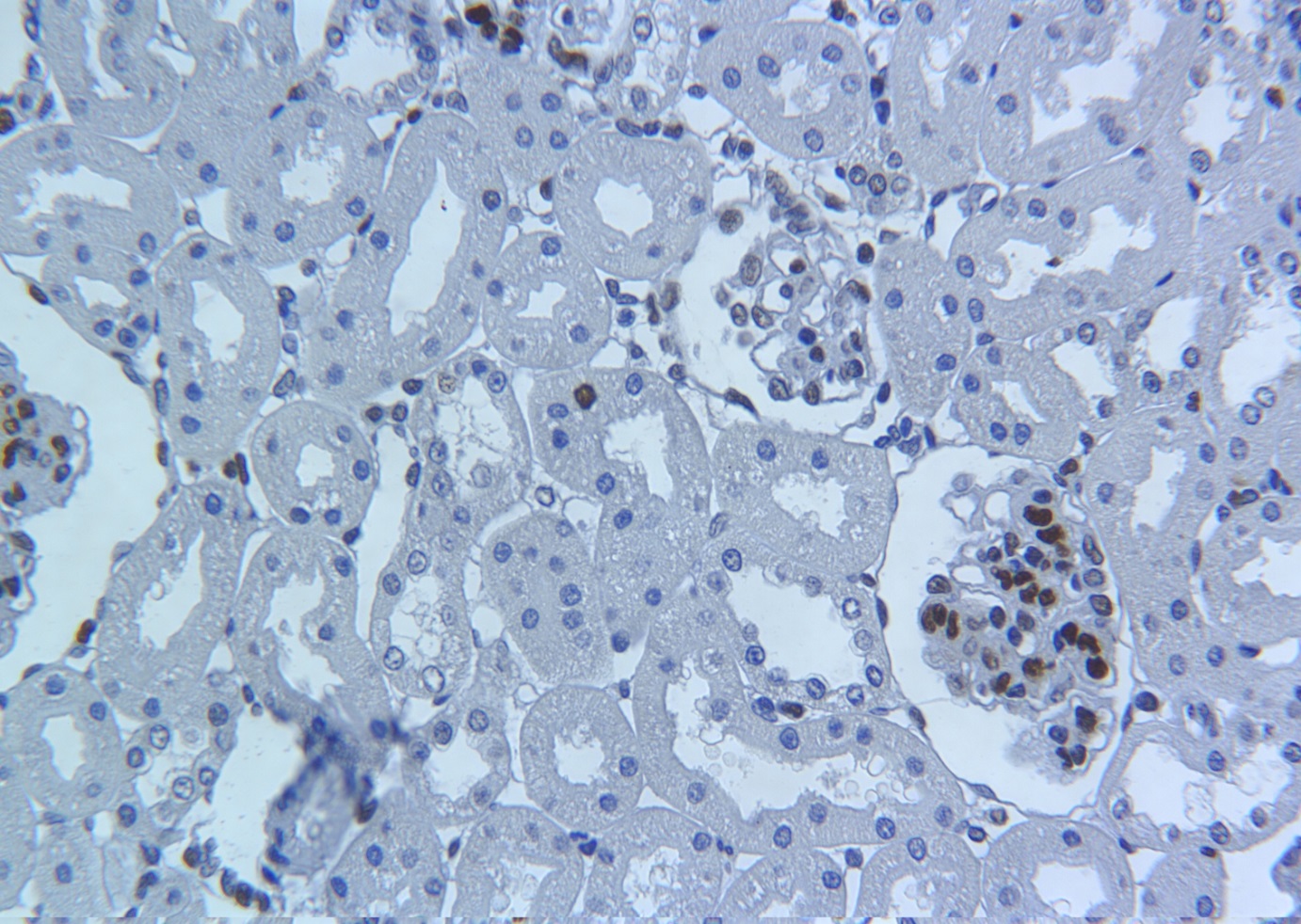


***Fig 6:*** Representative PCNA staining in rats suffering from mesangioproliferative Thy1.1 nephritis induced by anti thy1.1 monoclonal antibodies (upper panel) and nephritic animals pretreated with 5 mg/kg aprepitant i.p. 12h and 1h before injection of anti-Thy1.1 and every 24h after antibody challenge (lower panel). Seven days after the inducting of mesangioproliferative nephritis PCNA staining was significantly reduced in animals pretreated with aprepitant.

**Section D: Additional Material on TNF-α**

***Western Blot TNF α***


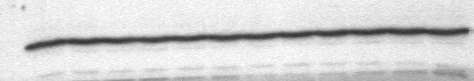

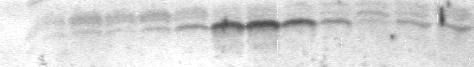


aprepitant

+ anti thy-1.1

anti thy-1.1

control

*Fig 7:* Western Blot for TNF α in controls, anti thy 1.1 nephritic rats and nephritic rats pretreated with aprepitant.
